# Associations between Harmful Experiences from Alcohol Use of Others and Mental Health in Korean Adolescents

**DOI:** 10.3390/ijerph16214240

**Published:** 2019-11-01

**Authors:** Mi Ah Han

**Affiliations:** Department of Preventive Medicine, College of Medicine, Chosun University, 309 Philmun-Daero, Dong-Gu, Gwangju 61452, Korea; mahan@chosun.ac.kr

**Keywords:** adolescent health, alcohol drinking, cross-sectional studies, health surveys, mental health, risk factors

## Abstract

This study assessed alcohol-induced harm to those not drinking and investigated its association with mental health in Korean adolescents. The 14th Korea Youth Risk Behavior Survey (2018) was used and 60,040 adolescents were analyzed. Harm from others’ alcohol consumption was assessed by four criteria: teasing in public places, being scared in public places, sleep problems, and unsafety of public places due to people drinking alcohol. Mental health included stress, depressive symptoms, suicidal ideation, and attempted suicide. Harm reported due to the alcohol use of others was 5.8% for teasing in public places, 33.6% for being scared in public places, 5.6% for sleep problems, and 40.2% for unsafety of public places among total study participants. Experiences of teasing in public places due to people who drank alcohol were associated with an increased risk of stress, depressive symptoms, suicidal ideation, and suicide attempts. Similarly, experiences of being scared in public places, sleep problems, and unsafety of public places due to people who drank alcohol were associated with poor mental health. In the stratified analysis, alcohol-induced harm was associated with poor mental health in both non-drinkers and drinkers. Harmful experiences from others’ alcohol consumption were associated with poor mental health among Korean adolescents.

## 1. Introduction

Alcohol use is a major risk factor for disease burden and leads to substantial health loss worldwide. According to the Global Burden of Disease Study, 32.5% of people globally were current drinkers, alcohol use was the seventh leading risk factor for premature death and disability, and 2.8 million deaths were attributed to alcohol use in 2016 [1]. Similarly, a number of epidemiological studies have reported adverse effects from drinking alcohol on various health outcomes such as cardiovascular disease [2], cancer [3], and mental health [4,5]. In particular, adolescents drinking alcohol in developmental stages are susceptible to adverse effects of alcohol, including neurocognitive effects [6,7].

Harm from drinking alcohol is not restricted to drinkers, but also can affect others (also called secondhand harm). In a national survey of adult Australians, 70% of respondents were negatively affected by strangers’ drinking, such as nuisance, fear, or abuse, and 30% by acquaintances’ drinking, and young adults were more likely to report the majority of types of harm [8]. In a cross-sectional survey of Indian adolescents, 44.5% were exposed to harm from others’ drinking, and harm outside the family was more common than within the family [9]. The exposure to harm from other people’s drinking negatively affected mental health; exposure to harm from others’ drinking, such as family problems or financial problems, was associated with increased levels of depression and distress in US adults in the 2010 U.S. National Alcohol Survey [10]. Similarly, a cross-sectional survey investigating the New Zealand general population reported that exposure to heavy drinkers was associated with reduced personal wellbeing and poorer health status [11].

According to the Korea National Health and Nutrition Examination Survey, the alcohol use rate of Korean adults increased from 57.3% in 2007 to 62.1% in 2017, and the binge drinking rate (more than seven drinks for men and five drinks for women during one occasion) was 39.0% in 2017 [12]. In Korea, drinking is regarded as a part of daily life and a medium for developing personal relationships and promoting intimacy; this leads to tolerance for drunken behaviors from the self and others [13]. While alcohol’s harm to the health of the drinker has been well described, much less is known about alcohol’s harm to people other than the drinker. Descriptions of the experience of harm from others’ drinking and its association with mental health are limited among Korean adolescents. Therefore, this study investigated the prevalence of exposure to harm from others’ alcohol use, and the association between exposure to alcohol harm from others and mental health in Korean adolescents.

## 2. Materials and Methods

### 2.1. Data Source and Study Population

The Korea Youth Risk Behavior Survey (KYRBS) is an annual national cross-sectional survey that has assessed health behaviors among Korean adolescents since 2005. It is conducted by the Ministry of Education, Ministry of Health and Welfare, and the Korea Center for Disease Control and Prevention (KCDC). The KYRBS uses multi-stage cluster sampling designed to obtain a nationally representative sample of Korean adolescents. Sample schools are selected using stratified cluster sampling methods, and one classroom from each grade in the sample school is selected as the sample classroom. All students in the sampled classes are invited to participate [14]. The questionnaires are developed based on literature, expert consultations, and advisory committees, and have been shown to be reliable and valid [14,15]. The response category is classified according to the definition of health indicators of KYRBS. In the 14th KYRBS (2018), 400 middle schools and 400 high schools totaling 62,823 students were selected and 60,040 participated; the participation rate was 95.6%. Informed consent was obtained for all participants and health information was collected using anonymously self-administered questionnaires. The institutional review board of the KCDC approved the protocols for the KYRBS. A detailed explanation is available elsewhere [14].

### 2.2. General Characteristics

General characteristics included sex (boys, girls), school year (middle 1st, middle 2nd, middle 3rd, high 1st, high 2nd, high 3rd), perceived school record (high, middle, low), family structure (lives with both parents, lives with father, lives with mother, lives with others), education level of parents (≤high school, ≥college, does not know/missing), perceived economic status (high, middle, low), currently smoking (no, yes), and physical activity (no, yes). Education level of parents was defined as the higher education level of the father and mother. Current smokers were defined as individuals who smoked on more than 1 day in the past 30 days. Physical activity was defined as exercise that increased the heart rate and made breathing difficult, regardless of exercise type, for more than five days, with a total of more than 60 min per day in the past 7 days.

Alcohol-related characteristics included current alcohol drinking (none, non-problematic drinking, problematic drinking), recommendation of alcohol drinking (no, yes), and education on alcohol drinking in school (no, yes). Current alcohol drinkers were defined as individuals who drank at least one alcoholic drink in the past 30 days. Among current drinkers, problematic alcohol drinking was defined as those who drank more than five drinks for boys and three drinks for girls, or those who lost consciousness and could not remember events in the past 30 days. Recommendation of alcohol drinking was defined as parents or relatives allowing the student to drink alcohol. Education on alcohol use was defined as having educational experience about alcohol in school in the past 12 months.

### 2.3. Harmful Experiences from Others’ Drinking

Harms from others’ drinking were assessed by four criteria, including teasing in public places, being scared in public places, sleep problems, and unsafety of public places due to people who drank alcohol. Experience was assessed using the question: “Have you ever experienced the following due to alcohol drinking of others in the past 12 months?” Each experience was defined as follows: (1) teasing in public places: I was teased or bothered by people who drank alcohol in the street or public places, (2) being scared in public places: I felt scared when I met people on the street who had been drinking alcohol, (3) sleep problems: I could not sleep at night due to people who had been drinking alcohol, and (4) unsafety of public places: I thought that public places were not safe due to people who had been alcohol. Each question was answered with “yes”, “no”, or “do not remember”, which were categorized into “yes” or “no” (“do not remember” was combined with “no”).

### 2.4. Mental Health

Mental health included stress, depressive symptoms, suicidal ideation, and suicide attempts. Stress was defined as responses of “very much” or “much” to the question: “How much do you feel stress in your usual life?”. Depressive symptoms were defined as a “yes” response to the question: “Did you feel so sad or hopeless almost every day for two weeks or more in a row that you stopped doing usual activities at any point in the past 12 months?” Suicidal ideation was defined as a “yes” response to the question: “Have you seriously thought of suicide at any point in the past 12 months?” A suicide attempt was defined as a “yes” response to the question: “Have you attempted suicide in the past 12 months?”

### 2.5. Analysis

All statistical analyses were performed using SAS version 9.4 (SAS Institute, Cary, NC, USA), taking into account the complex multi-stage sampling methods. PROC SURVEYFREQ using the logit method was used to calculate the proportions and 95% confidence interval (CI) of each experience of harm from others’ alcohol drinking by general characteristics of the adolescents. Multiple logistic regression analyses using PROC SURVEYLOGISTIC were conducted to examine the associations between harm experienced from others’ drinking and stress, depressive symptoms, suicidal ideation, and suicide attempts. Finally, stratified analysis by current alcohol drinking status was conducted to evaluate the association between harm experienced from others’ drinking and mental health. *p* < 0.05 was considered statistically significant.

## 3. Results

### 3.1. Experiences of Harms from Others’ Alcohol Drinking

Overall, 5.8% adolescents responded they had been teased or bothered by people who drank alcohol in the street or public places, and 33.6% felt scared when they met people on the street who had been drinking alcohol. In addition, 5.6% had been unable to sleep at night due to people who had been drinking alcohol, 40.2% had thought that public places were not safe due to people who had been drinking alcohol, and 49.8% reported one or more of these experience. Girls were more likely to report all four types of experience of harm from others’ drinking. While experiences of teasing in public places and sleep problems were more reported in problematic alcohol drinkers, being scared and feeling unsafe in public areas were more reported in non-drinkers (Table 1).

### 3.2. Association between Experiences of Harm from Others’ Alcohol Drinking and Mental Health

The prevalence of mental health problems were 40.4% stress, 27.1% depressive symptoms, 13.3% suicidal ideation, and 3.1% suicide attempts. Compared to unharmed adolescents, adolescents who were teased or bothered by people who drank alcohol in public places were more likely to report stress (adjusted odds ratio (aOR) = 1.95, 95% CI = 1.80–2.12), depressive symptoms (aOR = 1.87, 95% CI = 1.71–2.04), suicidal ideation (aOR = 1.87, 95% CI = 1.69–2.07), and suicide attempt (aOR = 2.24, 95% CI = 1.92–2.60). Similarly, experiences of being scared in public places, sleep problems, feeling unsafe in public places, and any experience were associated with increased risks of stress, depressive symptoms, suicidal ideation, and suicide attempts (Table 2).

### 3.3. Association between Experiences of Harm from Others’ Alcohol Drinking and Mental Health by Alcohol Drinking Status of Study Participants

Experience of harm from others’ drinking was significantly associated with poor mental health in both current and non-drinkers. In non-drinkers, teasing in public places was associated with stress (aOR = 2.21, 95% CI = 2.02–2.42), depressive symptoms (aOR = 1.92, 95% CI = 1.73–2.13), suicidal ideation (aOR = 1.87, 95% CI = 1.65–2.11), and suicide attempts (aOR = 2.31, 95% CI = 1.91–2.78). In addition, being scared in public places, sleep problems, feeling unsafe in public places, and any experience were associated with poor mental health among non-drinkers. Similarly, an experience of harm from others’ drinking was associated with stress, depressive symptoms, suicidal ideation, and suicide attempts among current alcohol drinkers (Table 3).

## 4. Discussion

This study explored the experience of harm from others’ drinking alcohol and examined its association with mental health status in Korean adolescents. Approximately 6% of adolescents experienced teasing in public places and 40% thought public places were unsafe because of people who drank alcohol. Each experience of harm from others’ drinking was associated with an increased risk of stress, depressive symptoms, suicidal ideation, and suicide attempts among Korea adolescents.

In previous studies of children and adolescents, experience rates of harm from others’ drinking have been varied by country, age group, types of harm, and measurement methods. Overall, 7.4% of caregivers reported harm to a child from someone drinking in the past year, including physical harm, yelling, lack of supervision, financial problems, or violence in the 2015 US National Alcohol’s Harm to Others Survey [16]. A representative national survey of New Zealanders found that 17% of respondents with children in the household reported that the children had been negatively affected by the drinking of someone else in the last 12 months; 11% were yelled at, criticized, or verbally abused, 7% had witnessed serious violence in the home, and 2% were physically hurt [17]. Approximately 44.5% Indian adolescents reported harm due to others’ alcohol use; 15.7% reported frequent harm, 43.3% psychological harm, 9.7% physical harm, 2.9% property harm, and 15.4% financial harm [9]. In this study, approximately 6% of adolescents reported that drinkers in public places had teased them, which was lower than in previous studies. This difference of experienced rate of harm could be interpreted as being due to methodological differences of the studies, and changing cultural attitudes towards alcohol drinking in the community. The questionnaire discussed in this study focused on public places and did not include the places where adolescents spend most of their time, such as home, school, and private academies [18], and did not include harm from people they knew, such as family, friends, or teachers. In previous studies, the home has been shown to be the most common place where harm from drinking occurs, and known people such as family and neighbors are the most common perpetrators of alcohol-related harm [9]. Therefore, due to the lack of this information, experiences of alcohol-induced harm from others might have been underestimated, and further studies taking into account where Korean adolescents spend their time are needed.

Among the four types of harm, unsafety of public places due to people who drank alcohol was the most highly reported at 40%. A recent nationwide cross-sectional study with 3015 adults aged 19 to 60 years reported that 57.3% of all participants and 71.2% of current drinkers had drunk alcohol in public places [19]. In Korea, there is no law prohibiting drinking in a public place, people can easily access alcohol at a grocery or convenience store, and people are tolerant toward drinking behaviors [13].

Differences of experiences of alcohol-induced harm according to general characteristics were observed, similar to previous studies. Girls were more likely to respond that they had experienced harm from others’ drinking in all four types of harm. Similarly, in previous studies with university students, female [20,21] students were more likely to experience alcohol-induced harm from others. Previous studies have explained that males might be more tolerant of disruptive alcohol-related behavior [21] and more familiar with non-bodily harm than females [20]. It could also be interpreted on the basis of gender differences in attitude toward alcohol drinking in Korea. Traditionally, alcohol drinking has been regarded as male behavior and as essential to social activities for men, whereas women have expected to be excluded from alcohol dinking culture. Although women have increased their social activities and chances to drink alcohol, gender differences still exist in drinking culture [22,23]. Men might be considered generous for drinking or becoming drunk in public places, whereas women might not. This gender difference might influence the reactions to alcohol drinking of others.

The drinking behavior of adolescents was associated with experiences of alcohol-induced harm from others; problematic drinkers were more likely to experience teasing in public places (10.8% vs. 5.1%) and sleep problems (7.8% vs. 5.3%), whereas non-drinkers were more likely to experience being scared (34.7% vs. 27.3%) and feeling unsafe in public areas (41.7% vs. 30.6%). In previous studies, drinkers reported more alcohol-related harm than non-drinkers [21], and problematic drinkers were more likely to be victims of another’s drinking [20,24]. Drinkers likely have more friends who drink than non-drinkers [25] and are more likely to be victims of their exposure to an alcohol-drinking environment, but they might also become accustomed to drinking or drunk people.

A total of 38.2% of adolescents reported that their family recommended or allowed alcohol drinking, and the proportions of harm experienced were higher in these adolescents in this study. Although this study did not directly investigate the alcohol-drinking status of family members, alcohol-drinking recommendations would suggest that family members drink or have a tolerant attitude toward drinking alcohol. It is true that adult-supervised settings for alcohol use resulted in higher levels of harmful alcohol consequences [26]. College students living in substance-free housing where neither alcohol nor smoking are allowed were less likely to experience secondhand effects of alcohol use [27]. In addition, adolescents who live in alcohol-free housing are more likely to be non-drinkers [28,29]. In particular, family-level factors are regarded as an important factor for adolescents’ drinking behaviors in Korea, and parents’ attitudes and behaviors for drinking could affect adolescents’ behaviors and attitudes [30]. This implies that living environments such as an alcohol-free residence or zone may protect students from direct and indirect harm from drinking alcohol.

Consistently with previous studies [10,11], experiences of harm from others’ drinking were associated with poorer mental health for Korean students. Experiencing teasing or sleep problems due to someone’s drinking may directly increase stress and depressive moods [31], and severe traumatic events may result in suicidal ideation or attempts [32]. Alternatively, because this study was cross-sectional, adolescents who have a mental health problem might perceive and report their experiences differently [10]. For example, people drinking alcohol on the street might be particularly scary for adolescents with depression, whereas other students might not be affected by the situation. In addition, experiences of harm from others’ drinking were associated with poor mental health after stratified analysis with alcohol-drinking status.

This study had limitations due to its use of the KYRBS. First, the KYRBS had a cross-sectional design and used the last 12 months as a reference period for reporting harm, which limits any conclusions related to a temporal and long-term relationship between alcohol’s harm and mental health. Adolescents who have mental problems might have a lower threshold for harmful experiences and have greater safety concerns in public places compared to adolescents without mental problems. To investigate long-term effects and causality, a longitudinal design is recommended. Second, this study could not investigate whether the person who drank alcohol was a stranger or a known person such as a relative. However, respondents were more likely to have reported the experience of harm by a stranger than by those they knew because the questions asked about their experiences in public places or the street, rather than home or school. Some studies have reported that while harmful exposure from strangers is more common than from acquaintances, adverse effects from known drinkers are generally more serious than those from strangers [8]. Therefore, further study is needed to assess the harm related to the relationship with the drinkers. Finally, the harm experiences of this study could have been due to the experience of someone who respondents knew, or harms that occurred in the respondents’ living areas, rather than respondents’ direct experiences. The measurement of harm might also be vague and imprecise because this study assessed exposure to harm using subjective questionnaires, which might depend on the threshold of respondents, and could not investigate frequency, duration, or severity of harm. Therefore, girls were more likely to experience harm form others’ drinking, which could be interpreted as their having a lower threshold compared to other adolescents. If further studies evaluate harm using quantitative indicators such as type of harm, frequency, duration, and severity, this harm could be measured more precisely [33].

## 5. Conclusions

Using a nationally representative study, experiences of harm from others’ alcohol drinking were associated with mental health in Korean adolescents. Alcohol drinking was negatively associated with surrounding people’s mental health, as well as the health of drinkers.

## Figures and Tables

**Table 1:** Experiences of alcohol-induced harm from others in Korean adolescents.

Characteristics	Number of Participants (%)	I was Teased or Bothered in the Street or Public Places by People Who Had Been Drinking Alcohol	I Felt Scared When I Met People on the Street Who Had Been Drinking Alcohol	I Could Not Sleep at Night due to People Who Had Been Drinking Alcohol	I Thought that Public Places were Not Safe Due to People Who Had Been Drinking Alcohol	Any Experience ^a^
Total	60,040 (100.0)	5.8 (5.6–6.1)	33.6 (32.3–34.9)	5.6 (5.4–5.8)	40.2 (39.1–41.3)	49.8 (48.5–51.1)
Sex						
Boys	30,463 (52.1)	3.9 (3.6–4.2)	14.7 (14.2–15.2)	4.7 (4.5–5.0)	24.6 (24.0–25.2)	31.4 (30.7–32.1)
Girls	29,577 (47.9)	7.9 (7.5–8.3)	54.1 (53.2–55.0)	6.6 (6.3–7.0)	57.1 (56.2–58.0)	69.7 (68.8–70.6)
School year						
Middle 1st	9847 (14.5)	2.8 (2.4–3.1)	31.5 (30.1–32.9)	4.1 (3.7–4.5)	37.4 (36.1–38.9)	47.8 (46.2–49.3)
Middle 2nd	10,092 (15.7)	3.9 (3.4–4.3)	30.4 (28.9–31.9)	5.1 (4.6–5.6)	36.7 (35.3–38.2)	46.7 (45.0–48.3)
Middle 3rd	10,290 (16.3)	5.2 (4.7–5.7)	32.6 (30.9–34.3)	5.9 (5.4–6.5)	38.8 (37.3–40.4)	48.3 (46.5–50.0)
High 1st	9260 (15.9)	6.6 (6.0–7.3)	34.0 (31.7–36.5)	5.9 (5.4–6.5)	41.4 (39.3–43.5)	51.1 (48.7–53.5)
High 2nd	10,039 (17.8)	7.1 (6.5–7.8)	35.0 (32.5–37.6)	6.1 (5.5–6.6)	41.1 (38.9–43.4)	50.3 (47.8–52.8)
High 3rd	10,512 (19.9)	8.2 (7.6–8.9)	36.8 (34.3–39.3)	6.3 (5.8–6.8)	44.4 (42.2–46.6)	53.3 (51.0–55.7)
Perceived school record						
High	23,420 (38.8)	6.4 (6.0–6.8)	36.6 (35.2–38.0)	5.7 (5.4–6.1)	42.0 (40.8–43.2)	52.1 (50.7–53.5)
Middle	17,526 (29.4)	5.1 (4.8–5.6)	33.8 (32.3–35.4)	5.2 (4.9–5.6)	40.9 (39.5–42.3)	50.5 (49.0–52.1)
Low	19,094 (31.9)	5.7 (5.3–6.1)	29.7 (28.3–31.1)	5.8 (5.5–6.2)	37.4 (36.2–38.7)	46.2 (44.8–47.7)
Family structure						
Lives with both parents	50,354 (84.6)	5.3 (5.1–5.6)	33.7 (32.4–35.0)	5.3 (5.1–5.5)	40.3 (39.2–41.5)	49.8 (48.5–51.1)
Lives with father	2444 (3.8)	7.4 (6.3–8.6)	28.4 (26.2–30.6)	6.4 (5.4–7.5)	36.7 (34.4–39.1)	46.4 (43.9–48.9)
Lives with mother	5591 (9.1)	7.6 (6.9–8.5)	35.8 (33.9–37.8)	6.9 (6.2–7.7)	42.3 (40.4–44.2)	52.9 (50.9–54.9)
Lives with others	1651 (2.5)	13.2 (11.3–15.3)	29.6 (26.9–32.5)	11.4 (9.6–13.4)	35.1 (32.4–37.8)	43.3 (40.3–46.3)
Education level of parents						
≤High school	13,885 (22.7)	6.4 (5.9–6.9)	34.3 (32.5–36)	6.4 (6.0–6.9)	41.3 (39.8–42.8)	51.0 (49.2–52.7)
≥College	36,836 (62.8)	5.9 (5.5–6.2)	35.6 (34.2–37)	5.3 (5.1–5.6)	42.0 (40.8–43.2)	51.8 (50.4–53.2)
Did not know/missing	9318 (14.5)	4.6 (4.2–5.2)	23.8 (22.6–25)	5.7 (5.1–6.2)	30.8 (29.5–32.1)	39.0 (37.5–40.5)
Perceived economic status						
High	24,207 (40.8)	5.5 (5.1–5.8)	32.3 (30.9–33.6)	4.8 (4.6–5.2)	38.6 (37.4–39.8)	47.8 (46.5–49.1)
Middle	27,808 (46.0)	5.3 (5.0–5.6)	33.7 (32.3–35.2)	5.2 (4.9–5.5)	40.6 (39.4–41.9)	50.2 (48.8–51.6)
Low	8025 (13.2)	8.6 (7.9–9.4)	37.0 (35.1–38.9)	9.5 (8.8–10.2)	43.7 (41.9–45.6)	54.2 (52.3–56.2)
Currently smoking						
No	56,318 (93.3)	5.5 (5.3–5.8)	34.7 (33.4–36.0)	5.5 (5.3–5.8)	41.4 (40.3–42.5)	51.0 (49.7–52.3)
Yes	3722 (6.7)	9.6 (8.6–10.8)	18.5 (16.9–20.1)	6.7 (5.8–7.6)	23.7 (22.1–25.4)	32.6 (30.7–34.6)
Physical activities						
No	8585 (13.9)	5.5 (4.9–6.1)	21.3 (20.2–22.5)	5.3 (4.8–5.9)	31.1 (29.8–32.3)	38.4 (37.1–39.7)
Yes	51,455 (86.1)	5.9 (5.6–6.1)	35.6 (34.2–36.9)	5.7 (5.4–5.9)	41.7 (40.5–42.9)	51.6 (50.3–52.9)
Current alcohol use						
None	50,373 (83.1)	5.1 (4.9–5.4)	34.7 (33.4–36.1)	5.3 (5.1–5.6)	41.7 (40.5–42.8)	51.1 (49.8–52.4)
Non-problematic drinking	4393 (7.6)	7.1 (6.3–8.0)	28.6 (26.7–30.6)	6.1 (5.4–6.9)	35.9 (34.0–37.9)	44.7 (42.6–46.9)
Problematic drinking	5274 (9.3)	10.8 (9.9–11.8)	27.3 (25.4–29.3)	7.8 (6.9–8.7)	30.6 (28.8–32.4)	41.6 (39.5–43.7)
Recommendation of alcohol use						
No	37,711 (61.8)	4.6 (4.3–4.9)	32.5 (31.2–33.7)	4.8 (4.6–5.1)	39.3 (38.2–40.4)	48.2 (47.0–49.5)
Yes	22,329 (38.2)	7.7 (7.3–8.2)	35.4 (33.8–37.0)	6.9 (6.5–7.3)	41.7 (40.4–43.1)	52.2 (50.7–53.8)
Education of alcohol use						
No	34,214 (58.0)	5.5 (5.2–5.9)	32.9 (31.4–34.4)	5.5 (5.2–5.8)	39.0 (37.7–40.3)	48.5 (47.0–50.0)
Yes	25,826 (42.0)	6.2 (5.8–6.5)	34.5 (33.2–35.8)	5.8 (5.5–6.1)	41.9 (40.7–43.1)	51.5 (50.2–52.8)

Data are expressed as % (95% confidence interval). ^a^ Includes any respondent reporting one or more of the four harm categories.

**Table 2:** Associations between experience of alcohol-induced harm and mental health in Korean adolescents.

Due to People Who Had Been Drinking Alcohol	Stress		Depressive Symptoms		Suicidal Ideation		Suicide Attempt	
	%	OR (95% CI) ^a^	%	OR (95% CI) ^b^	%	OR (95% CI) ^c^	%	OR (95% CI) ^c^
Total	40.4		27.1		13.3		3.1	
Teased in public places								
No	39.2	1.00	25.8	1.00	12.3	1.00	2.7	1.00
Yes	60.8	1.95 (1.80–2.12)	48.6	1.87 (1.71–2.04)	30.6	1.87 (1.69–2.07)	9.7	2.24 (1.92–2.60)
Scared in public places								
No	34.7	1.00	22.3	1.00	10.6	1.00	2.5	1.00
Yes	51.8	1.62 (1.55–1.69)	36.5	1.54 (1.47–1.63)	18.8	1.24 (1.15–1.33)	4.2	1.21 (1.07–1.36)
Sleep problems								
No	39.4	1.00	26.0	1.00	12.5	1.00	2.7	1.00
Yes	57.7	1.86 (1.72–2.01)	45.1	1.78 (1.64–1.94)	27.0	1.60 (1.44–1.77)	9.1	2.17 (1.86–2.53)
Unsafety of public places								
No	34.3	1.00	22.5	1.00	10.7	1.00	2.6	1.00
Yes	49.5	1.58 (1.52–1.64)	34.0	1.40 (1.33–1.47)	17.3	1.18 (1.10–1.26)	3.8	1.14 (1.01–1.29)
Any experience ^d^								
No	32.4	1.00	20.7	1.00	9.7	1.00	2.3	1.00
Yes	48.5	1.62 (1.56–1.68)	33.5	1.50 (1.43–1.58)	17.0	1.21 (1.14–1.30)	3.8	1.21 (1.06–1.37)

CI, confidence interval; OR, odds ratio. ^a^ adjusted for sex, school year, perceived school record, family structure, education level of parents, perceived economic status, place of residence, current smoking status, physical activities, current alcohol use, recommendation of alcohol use, and alcohol use education; ^b^ additionally adjusted for stress; ^c^ additionally adjusted for depressive symptoms; ^d^ includes any respondent reporting one or more of the four harm categories.

**Table 3:** Association between experiences of alcohol-induced harm and mental health by alcohol use status.

Characteristics	Non-Drinkers	Non-Problematic Drinkers	Problematic Drinkers
Stress ^a^			
Teased in public places	2.21 (2.02–2.42)	1.81 (1.37–2.38)	1.11 (0.91–1.35)
Scared in public places	1.66 (1.59–1.74)	1.60 (1.36–1.88)	1.26 (1.08–1.46)
Sleep problems	2.00 (1.83–2.19)	1.77 (1.33–2.36)	1.13 (0.89 –1.42)
Unsafety of public places	1.62 (1.55–1.69)	1.46 (1.27–1.68)	1.33 (1.15–1.53)
Any experience ^b^	1.68 (1.60–1.75)	1.43 (1.25–1.64)	1.35 (1.18–1.54)
Depressive symptoms ^c^			
Teased in public places	1.92 (1.73–2.13)	1.45 (1.11–1.89)	1.75 (1.43–2.13)
Scared in public places	1.58 (1.49–1.67)	1.46 (1.21–1.74)	1.29 (1.10–1.51)
Sleep problems	1.72 (1.57–1.89)	1.99 (1.48–2.66)	1.82 (1.41–2.34)
Unsafety of public places	1.44 (1.37–1.52)	1.30 (1.11–1.53)	1.14 (0.99–1.33)
Any experience ^b^	1.55 (1.47–1.64)	1.45 (1.23–1.70)	1.21 (1.05–1.40)
Suicidal ideation ^d^			
Teased in public places	1.87 (1.65–2.11)	1.32 (0.96–1.83)	1.99 (1.56–2.54)
Scared in public places	1.20 (1.11–1.29)	1.31 (1.05–1.65)	1.32 (1.08–1.63)
Sleep problems	1.53 (1.35–1.73)	1.38 (0.94–2.03)	1.91 (1.45–2.52)
Unsafety of public places	1.15 (1.07–1.24)	1.11 (0.89–1.39)	1.32 (1.10–1.58)
Any experience ^b^	1.18 (1.10–1.28)	1.20 (0.96–1.48)	1.36 (1.14–1.62)
Suicide attempt ^d^			
Teased in public places	2.31 (1.91–2.78)	1.23 (0.76–1.99)	2.35 (1.75–3.16)
Scared in public places	1.11 (0.96–1.28)	1.09 (0.74–1.62)	1.49 (1.13–1.97)
Sleep problems	2.01 (1.66–2.43)	2.02 (1.16–3.52)	2.47 (1.78–3.41)
Unsafety of public places	1.07 (0.92–1.23)	0.97 (0.71–1.33)	1.43 (1.09–1.88)
Any experience ^b^	1.13 (0.96–1.32)	0.93 (0.66–1.30)	1.61 (1.21–2.13)

Data are expressed as adjusted odds ratio (95% confidence interval). ^a^ adjusted for sex, school year, perceived school record, family structure, education level of parents, perceived economic status, current smoking status, physical activities, recommendation of alcohol use, and alcohol education; ^b^ includes any respondent reporting one or more of the four harm categories; ^c^ additionally adjusted for stress; ^d^ additionally adjusted for depressive symptoms.
